# Adaptation and Validation of the Malay-Chrononutrition Profile Questionnaire to Assess Chrononutrition Behavior of Young Adults in Malaysia

**DOI:** 10.1016/j.cdnut.2022.100009

**Published:** 2022-12-22

**Authors:** Khairunnisa Fazira Hairudin, Nur Islami Mohd Fahmi Teng, Norsham Juliana

**Affiliations:** 1 Faculty of Health Sciences, Universiti Teknologi MARA, Bandar Puncak Alam, Malaysia; and; 2 Faculty of Medicine and Health Sciences, Universiti Sains Islam Malaysia, Nilai, Malaysia

**Keywords:** chrononutrition, young adults, Malaysia, Malay-CPQ, validation

## Abstract

**Background:**

Chrononutrition studies how biological rhythms and nutrition are associated with human health. However, a validated assessment in Malaysia is still absent.

**Objectives:**

To create a translation of the Chrononutrition Profile Questionnaire (CPQ), test its validity and reliability, and determine the general chrononutrition behaviors among Malaysian young adults.

**Methods:**

The Malay-CPQ was distributed to respondents through online platforms (*n* = 110), and data analyses were performed. The data were analyzed for their validity using content validity index (CVI) and face validity index (FVI), whereas intraclass correlation coefficient (ICC) was used to determine test–retest reliability.

**Results:**

Our results showed both CVI and FVI of Malay-CPQ were 1, indicating excellent content translation, while the ICC values ranged from moderate to good (0.50–0.90). The Cronbach α values for all items ranged from moderate to good (0.50–0.90), and the Bland–Altman analysis showed a *P* value >0.05, indicating agreement of the item between repeated measurements. The chrononutrition behaviors among Malaysian young adults presented fair to good scores for all behavior patterns: eating window, breakfast skipping, evening eating, night eating, and largest meal, except evening latency, being mostly at the poor score (>80% responses).

**Conclusions:**

The Malay-CPQ is a valid and reliable tool to assess the Malaysian chrononutrition profile. However, further testing on Malay-CPQ should be conducted in a different setting in Malaysia for cross-validation studies.

## Introduction

Chrononutrition is defined as the study of biological rhythms and nutrition and its relationship with human health, which include energy distribution, meal frequency, and regularity [1]. It is an emerging study on how food affects the body’s circadian rhythm [2]. Six distinct behavioral patterns are likely to influence one’s chrononutrition: *1*) eating at night, *2*) restricted time feeding, *3*) breakfast eating, *4*) timing of the largest meal, *5*) time of evening eating, and *6*) time between eating and sleep time [3]. The circadian mealtime pattern is essential for food intake regulation and body metabolism to ensure the well-being status of an individual is optimum. It was reported that poor adherence to a healthy diet was usually exhibited by individuals with late chronotype [4, 5]. Moreover, this group observed a habit of delaying the timing of eating, frequently skipping breakfast, showing a high preference for sweet food and drinks, drinking alcohol, and not consuming enough fruit and vegetables. Unfortunately, individuals who have a high-calorie intake at a later time of the day are exposed to the risk of getting obese [6, 7]. This is because the process of lipogenesis and accumulation of adipose tissues tend to occur during the period of the last meal. Additionally, breakfast skippers will experience a longer period of lipolysis and lipogenesis owing to their eating habits [8]. These unhealthy eating habits eventually will affect the wellness of an individual, in the aspects of cognitive, behavioural, and physical status. Disruption in circadian rhythm will result in the development of morbidities or metabolic diseases such as abdominal obesity, dyslipidemia, dysglycemia, and hypertension. Thus, chrononutrition may affect the quality of life of an individual owing to the misalignment interaction between dietary intake and meal timing. A study by Oike et al. [9] highlighted that many countries with a high prevalence of metabolic diseases recorded people with disrupted mealtimes and deprived sleep. In addition, chronodisruption or loss of circadian pattern causes a decline in health and exposes one to metabolic abnormalities. Eating late at night was reported to increase the risk of getting obese [10]; whereas, skipping breakfast was found to affect the concentration of postprandial insulin and might disrupt normal glucose homeostasis [4].

The Chrononutrition Profile Questionnaire (CPQ) was introduced by Veronda et al. [3], which was developed and validated in the United States. Generally, CPQ has been designed as a comprehensive assessment of chrononutrition aspects without burdening participants. Consisting of 18 items, CPQ is intended to determine patterns of chrononutrition behaviors and chrononutrition preferences on both weekdays and weekend days. There are 4 domains of the CPQ, which asks about the following: *1*) chrononutrition preferences; *2*) frequency of certain chrononutrition behavior; *3*) timing of eating events during workday; and *4*) timing of eating events during a free day. The first and second domains consist of 4 items while the third and fourth 5 items each. Because chrononutrition assessment is still new, having a specific and validated tool to assess meal timings and patterns is necessary.

Interventions through chrononutrition profile assessment as a recent and different approach for health management shall help improve an individual's quality of life. However, a validated tool is absent in Malaysia assessing chrononutrition profiles. Thus, this study aimed to create a translation of the CPQ into the Malay language and to test its reliability and validity among young adults in Malaysia. This is necessary because the first language in Malaysia is Malay; hence, a Malay-CPQ is desired to benefit other generations or populations in Malaysia who may not be fluent in English.

## Materials and Methods

This study was a quantitative and cross-sectional study that involved 4 stages of validation: *1*) Forward and backward translation was conducted by 2 bilingual translators, translating from the English version of CPQ to the Malay language and back to the English language by another 2 bilingual translators. All translators hold a higher degree in education and have at least 2 years of experience in translation and language teaching. *2*) An expert committee review and content validity was performed by a panel of 6 experts. The panel comprised nutritionists, dieticians, researchers, and academics who are proficient in English and Malay language and understand the construct of CPQ. They were asked to rate on a 4-point Likert scale: score 1 (item very not relevant) to 4 (item very relevant), based on the items’ relevancy to the Malay-CPQ. Scores 3 and 4 were reclassified as 1 (relevant), whereas scores 1 and 2 were recategorized as 0 (not relevant). *3*) pilot testing was conducted with 10 bilingual undergraduate students who rated and commented on the items. *4*) A reliability test through test–retest reliability (*n* = 110) was performed. Respondents were recruited through various online platforms such as WhatsApp, Twitter, and Instagram. On consent, the questionnaires were distributed online and were administered twice, within 14–21-d intervals. The targeted respondents were Malaysian undergraduate students aged 18–30 y who understood the Malay language. In the pilot study, respondents were asked to complete the questionnaire and rate on a 4-point Likert scale of 1 (item very not clear and understandable) to 4 (item is very clear and understandable). Scores 3 and 4 were reclassified as 1 (clear and understandable) and scores 1 and 2 as 0 (not clear and understandable). Young adult participants was chosen as the target group in this study as little is known about chrononutrition behavior of the young adults or college students and feasibility in the subject recruitment. Nonetheless, a recent study in Malaysia on the CPQ validation has focused on the adult population (aged 18–65 y). Therefore, this study aimed to provide a more specific target group to assess the reliability [11].

The content validity index (CVI) is an index used in quantitative evaluation. There are 2 types of CVI: I-CVI for chance agreement and S-CVI for average (S-CVI/Ave) and universal agreement (S-CVI/UA). All these have different formulas to determine the degree of item appropriateness to be measured in a questionnaire. A CVI score of >0.83 indicated that the items in the questionnaire were relevant to the domain. A scale of <0.79 indicates that the item requires revision, and if it was <0.70, the item should be eliminated. On the contrary, the face validity index (FVI) is an index to the extent of whether the items measure what it is intended to measure. Two types of FVI are I-FVI for chance agreement and S-FVI for average (S-FVI/Ave) and universal agreement (S-FVI/UA). Similar to CVI, all these have different formulas to measure item relevancy in questionnaires. The satisfactory level of FVI was taken at 0.83 or more, adopted from the CVI value. A scale of <0.79 indicated that the item requires revision, and if it was <0.70, the item should be eliminated [12].

Data obtained were analyzed by using IBM SPSS 20 involving several analyses. The first analysis was the intraclass correlation coefficient (ICC), intended to establish and quantify reproducibility and provide an indication of the test–retest reliability of measurement. The ICCs were calculated by using a 2-way, mixed consistency model, with an acceptable value of 0.50 as moderate reliability, 0.75–0.90 as good reliability, and >0.90 as excellent reliability [13]. Next, separated correlation coefficients were computed for weekdays and weekend values for chrononutrition behaviors to ensure the continuous values of items were reliable because those were the foundation of computed weekly average and chrononutrition behavior cutoff scores.

The second analysis used Cronbach α to assess the internal consistency of items with acceptable values ranging from 0.60 to 0.95 [14]. A value >0.95 might indicate the presence of redundant items. Then, the agreement of reported values was measured with the Bland–Altman analysis. The *P* value for each item should be >0.05 to reject the null hypothesis, indicating there was no proportional bias for the item between repeated measurements. As for chrononutrition behavior, data were classified according to scoring cutoffs for the chrononutrition profile derived from a table in the study by Engwall [15].

This study has obtained approval from the Universiti Teknologi MARA Research Ethics Committee, with a reference number REC/06/2021 (UG/MR/589).

## Results

### Content and face validity indices

Overall, the I-CVI, S-CVI/Ave, and S-CVI/UA scales obtained a score of 1, which was considered excellent, indicating that the content of translated Malay-CPQ version for each item was well adapted to the local context. A CVI score of >0.83 indicated that the items in the questionnaire were relevant to the domain. All comments given by the expert panels were taken to improve the comprehension of items. Table 1 presents detailed information on the CVI.

**Table 1:** The clarity and comprehension ratings on the items scale by 6 experts for content validity

Item	Expert 1	Expert 2	Expert 3	Expert 4	Expert 5	Expert 6	No. of agreements	I-CVI
A1	3	3	3	3	3	3	6	1
A2	3	3	3	3	3	3	6	1
A3	3	3	3	3	3	3	6	1
A4	3	3	3	3	3	3	6	1
B1	4	3	3	3	4	3	6	1
B2	4	3	3	3	4	3	6	1
B3	4	3	3	3	4	3	6	1
B4	4	3	3	3	4	3	6	1
C1	4	3	3	4	4	3	6	1
C2	4	3	3	4	4	3	6	1
C3	4	3	3	4	4	3	6	1
C4	4	3	3	4	4	3	6	1
C5	4	3	3	4	4	3	6	1
D1	4	3	3	4	3	3	6	1
D2	4	3	3	4	3	3	6	1
D3	4	3	3	4	3	3	6	1
D4	4	3	3	4	3	3	6	1
D5	4	3	3	4	3	3	6	1
							S-CVI/average	1
							Total agreement	18
							S-CVI/UA	1

CVI, content validity index; UA, universal agreement.

Generally, the I-FVI, S-FVI/Ave, and S-FVI/UA scales obtained a score of 1, which was considered excellent, indicating that the face validity of translated Malay-CPQ version for each item was using clear and understandable sentences. In addition, an FVI score of >0.83 indicated that the items in the questionnaire were relevant to the domain. In summary, item content and face validity had been proven to be valid, and the validated Malay-CPQ could be used for test–retest reliability purposes. Table 2 tabulates the detailed information on the FVI.

**Table 2:** The clarity and comprehension ratings on the items scale by 10 raters for face validity (pilot testing).

	Rater 1	Rater 2	Rater 3	Rater 4	Rater 5	Rater 6	Rater 7	Rater 8	Rater 9	Rater 10	No. of agreements	I-FVI
Item												
A1	4	4	3	4	4	4	4	3	4	4	10	1
A2	4	4	3	4	4	4	4	3	4	4	10	1
A3	4	4	3	4	4	4	4	3	4	4	10	1
A4	4	4	3	4	4	4	4	3	4	4	10	1
B1	3	4	3	4	3	3	4	3	4	3	10	1
B2	3	4	3	4	3	3	4	3	4	3	10	1
B3	3	4	3	4	3	3	4	3	4	3	10	1
B4	3	4	3	4	3	3	4	3	4	3	10	1
C1	4	4	4	4	4	4	4	4	4	4	10	1
C2	4	4	4	4	4	4	4	4	4	4	10	1
C3	4	4	4	4	4	4	4	4	4	4	10	1
C4	4	4	4	4	4	4	4	4	4	4	10	1
C5	4	4	4	4	4	4	4	4	4	4	10	1
D1	4	4	4	4	4	4	4	4	4	4	10	1
D2	4	4	4	4	4	4	4	4	4	4	10	1
D3	4	4	4	4	4	4	4	4	4	4	10	1
D4	4	4	4	4	4	4	4	4	4	4	10	1
D5	4	4	4	4	4	4	4	4	4	4	10	1
											**S-FVI/Ave**	1
											**Total Agreement**	18
											**S-FVI/UA**	1

FYI, face validity index; UA, universal agreement.

### Test–retest reliability

Reported chrononutrition preferences (e.g., wake time preference and preferred bedtime) were correlated (Table 3). Correlations for workday values ranged from moderate (wake time, first eating event, last eating event, and bedtime) to good (lunchtime), whereas correlations for free day values ranged from moderate (first eating event, lunchtime, and last eating event) to good (wake time and bedtime). Weekly averages were computed for eating window and after-dinner snacks, showing moderate correlations. In addition, correlations between evening eating, evening latency, and breakfast frequency were good.

**Table 3:** Values on test–retest reliability involving the ICC, Cronbach α, and Bland–Altman analysis

Item	Test (mean ± SD)	Retest (mean ± SD)	Cronbach α	ICC (average measure)	Bland–Altman
Chrononutrition preferences					
Wake time	7.72 ± 1.82	7.59 ± 1.71	0.90	0.90	0.255
Morning latency	1.74 ± 1.54	1.61 ± 1.30	0.86	0.86	0.008
Evening latency	2.78 ± 1.68	2.92 ± 1.69	0.79	0.79	0.916
Bedtime	7.18 ± 4.82	8.12 ± 4.62	0.72	0.72	0.600
Chrononutrition behaviors: weekdays					
Wake time	6.81 ± 1.13	7.01 ± 1.35	0.60	0.59	0.044
First eating event	8.34 ± 2.35	8.34 ± 2.36	0.64	0.64	0.943
Lunchtime	3.91 ± 4.47	3.65 ± 4.51	0.83	0.83	0.877
Last eating event	9.20 ± 1.38	9.02 ± 1.56	0.73	0.72	0.132
Bedtime	7.06 ± 5.09	7.22 ± 5.06	0.70	0.70	0.946
Chrononutrition behaviors: weekend days					
Wake time	8.54 ± 1.94	8.48 ± 2.14	0.76	0.76	0.209
First eating event	8.03 ± 3.56	8.45 ± 3.36	0.50	0.50	0.521
Lunchtime	3.35 ± 3.85	3.59 ± 4.05	0.62	0.62	0.560
Last eating event	9.36 ± 1.62	9.34 ± 1.57	0.70	0.70	0.728
Bedtime	5.76 ± 4.77	6.13 ± 4.93	0.85	0.85	0.638
Computed weekly averages					
Breakfast frequency	4.43 ± 2.21	4.74 ± 2.01	0.88	0.87	0.121
Evening eating	9.25 ± 1.37	9.14 ± 1.52	0.75	0.75	0.180
After-dinner snack	3.28 ± 1.95	3.21 ± 1.90	0.71	0.71	0.737
Evening latency	−2.56 ± 5.05	−2.22 ± 5.11	0.79	0.79	0.867
Eating window	1.00 ± 2.39	0.76 ± 2.50	0.70	0.70	0.594

All correlations were significant at the *P* < 0.001 level.

ICC, intraclass correlation coefficient.

Cronbach α was calculated to determine internal consistency. Reported chrononutrition preferences were relatively high in consistency. In addition, the internal consistency for the workday was high for lunchtime, last eating event, and bedtime and moderate for wake time and first eating event. Furthermore, the internal consistency for free days was high for wake time, last eating event, and bedtime, moderate for lunchtime but low for first eating event. Weekly averages were computed for breakfast frequency, evening eating, after-dinner snack, evening latency, and eating window, and the internal consistency was high. Table 3 provides detailed information on test–retest reliability involving ICC, Cronbach α, and Bland–Altman analysis values.

The item with a poor reliability coefficient (first eating event during free days) was checked for its extent of agreement with the Bland–Altman analysis. The *P* value of the first eating event for free days was >0.05, indicating the item could reject the null hypothesis and had no proportional bias. Therefore, all items were proven valid and reliable, allowing the final version of Malay-CPQ to consist of 18 items.

### Sociodemographic information

Most participants in this study were women. Each participant had a consistent demographic profile, with all of them being Malaysian citizens, Malay, and undergraduate students from various universities in Malaysia. The ages of participants ranged from 18 to 28 y (mean: 21.50 y; SD: 2.32 y). Most of the participants showed normal BMI values (51.8%), followed by underweight (19.1%) and overweight (11.8%) (Table 4).

**Table 4:** Sociodemographic details (*n* = 110)

Demographic variable	Parameter
Age (y)	
Mean	21.50
SD	2.32
Sex, *n* (%)	
Male	11 (10)
Female	99 (90)
BMI, *n* (%)	
Underweight	21 (19.1)
Normal	57 (51.8)
Overweight	19 (17.3)
Obese	13 (11.8)

### Chrononutrition and scoring cutoffs for the chrononutrition profile

Descriptive statistics were used to explore the characteristics of the chrononutrition profile. More specifically, this study examined the range and distributions of scores on individual items and chrononutrition behavior cutoff classifications. All participants reported responses in the Malay-CPQ test and retested the version excellently. Hence, this analyses included all 6 general chrononutrition behaviors. Table 5 summarizes detailed chrononutrition behavior cutoffs for chrononutrition profiles from Malay-CPQ (test) and Malay-CPQ (retest).

**Table 5:** Distribution of chrononutrition behavior cutoffs for Chrononutrition Profile from Malay-CPQ (test) and Malay-CPQ (retest) (*n* = 110)

Chrononutrition behavior cutoffs	Malay-CPQ (test), *n* (%)	Malay-CPQ (retest), *n* (%)
Eating window		
Good (≤12:00)	110 (100)	110 (100)
Fair (12:01–14:00)	—	—
Poor (>14:00)	—	—
Breakfast skipping		
Good (1 d/wk or less)	39 (35.5)	44 (40.0)
Fair (2–3 d/wk)	32 (29.0)	32 (29.0)
Poor (≥4 d/wk)	39 (35.5)	34 (30.9)
Evening latency		
Good (>6:00)	—	—
Fair (2:01–6:00)	24 (21.8)	25 (22.7)
Poor (≤2:00)	86 (78.2)	85 (77.3)
Evening eating		
Good (<20:00)	12 (10.9)	12 (10.9)
Fair (20:00–22:59)	85 (77.3)	86 (78.2)
Poor (≥23:00)	13 (11.8)	12 (10.9)
Night eating		
Good (1 d/wk or less)	94 (85.5)	90 (81.8)
Fair (2–3 d/wk)	9 (8.2)	14 (12.7)
Poor (≥4 d/wk)	7 (6.4)	6 (5.5)
Largest meal		
Breakfast	1 (0.9)	3 (2.7)
Lunch	91 (82.7)	83 (75.5)
Dinner/supper	18 (16.4)	24 (21.8)

Malay-CPQ, Malay-Chrononutrition Profile Questionnaire.

The eating window from both test and retest Malay-CPQ were 100% matched, and all participants exhibited a good duration between the first and last eating events (≤12:00). The reported breakfast skipping between the test and retest questionnaire showed that most of the participants had not or only skipped breakfast 1 dci, along a week, followed by more than one-third exhibiting poor habit by skipping ≥4 d/wk, and less than one-third reporting fair habit with skipping for 2–3 d/wk.

A poor outcome was reported for evening latency because almost 80% of participants reported a poor duration between the last eating event and sleep onset (≤2:00), followed by <30 of them having fair scores (2:01–6:00) for both test and retest Malay-CPQ. The evening eating, in which the risk of eating late in the waking day was reported, showed almost 80% of participants having a fair score (20:00–22:59) and <12% for both poor (≥23:00) and good (<20:00) scores. The reported frequency of night eating had obtained results with >80% of the participant having good scores (1 d/wk or less), <15% having fair (2–3 d/wk), and poor (≥4 d/wk) scores. Lunchtime was reported as the largest meal by the most of the participants for both test and retest Malay-CPQ (>75%), dinner/supper as the second-largest meal (>16%), and breakfast as the least one (>3%).

## Discussion

This present study aimed to develop and evaluate the Malay-CPQ: a measure derived from the original version, CPQ [15]. The Malay-CPQ assessed 6 specific behavioral patterns likely to affect one’s chrononutrition profile: *1*) eating window, *2*) breakfast skipping, *3*) evening latency, *4*) evening eating, *5*) night eating, and *6*) largest meal. Overall, the findings of this study corroborate the Malay-CPQ hypothesis.

CPQ was chosen as this study instrument because it consisted of comprehensive items to measure the chrononutrition pattern including time of eating event and sleep time that requires a short time for respondents to answer. The items were simple and did not give a significant burden on respondents. The original CPQ was translated to fit Malaysian culture because the first language in Malaysia is Malay. Compared with other tools measuring meal timing, such as the Food Timing Questionnaire (FTQ) and Food Timing Screener (FTS), the CPQ can capture the largest meal of the day and calculate eating misalignment as well [16]. The FTQ is designed to assess an individual’s usual eating time and sleep habits throughout the week, whereas FTS assesses the same elements but on separate types of day: school/work days and free days. FTS seemed to have the same construct as CPQ, which allows accurate food intake and mealtime during school/work days and free days.

The translated Malay-CPQ version provided valid and reliable results when it was applied. This study looked at content equivalence and semantic equivalent to ensure that the quality and consistency of the meaning in the translated version matched the original version. The translation and adaptation processes in this study conformed to the guidelines that the translated instruments need to be evaluated regarding content, conceptual, context, semantic, and technical equivalency to ensure the instrument is appropriate for use in the new setting. Failure to adhere to the guidelines might lead to significant contextual and conceptual equivalence issues.

In this study, overall, the individual items rated by 6 expert panels, the I-CVI scale obtained a score of 1 (relevant), which is considered excellent, indicating that the content of the translated Malay-CPQ version is well adapted to the local context. A CVI score of >0.8 indicates that the items in the questionnaire are relevant to the domain. The excellent score was supported with both S-CVI/Ave and S-CVI/UA scales, which obtained a score of 1. A score of 1 was the highest score that can be given to evaluate items. This score was a combination of scores 3 and 4 given by expert panels for each item and was classified on an acceptable scale of 1.

As for the individual items ranged by undergraduate students, overall, the I-FVI scale obtained a score of 1 (relevant) as well. This shows that the original CP-Q is well translated into the Malay language using clear and understandable sentences. The result was supported by both S-FVI/Ave and S-FVI/UA scales, which also obtained a score of 1. The satisfactory level of the FVI was taken at 0.8 and above, and it was adopted from the CVI values.

The Malay-CPQ provided strong evidence for test–retest reliability, with all items significantly correlating over the 14–21-d period. Two aspects should be noted regarding the test–retest reliability: *1*) weekend values were slightly less correlated compared with weekdays values, and 2) the correlation coefficient for the first eating event on weekend days was particularly low compared with that of the other items. This could indicate that the timing of food intake on weekend days may be more variable over time than the timing of food intake on weekdays, and the first eating event may vary more on weekends compared with the timing of other eating events. In addition, chrononutrition preferences were strongly correlated over the testing period. The data were collected within 2 mo, from September until October of 2021, whereby there were no new changes in the restriction orders due to the COVID-19 pandemic announced by the government of Malaysia during the period. Therefore, these strong correlations indicate that the Malay-CPQ is reliable based on the ICC values.

The internal consistency measured with Cronbach α values showed good consistency for all items except for the first eating event from the weekend value. The increased duration of online learning was reported to be associated with breakfast skipping and increased sugar-sweetened beverages during the COVID-19 restrictions [17]. In addition, the rising use of computers, tablets, and smartphones for online learning has been linked to poor sleep duration and lower quality of life regarding mental health. These had affected students’ wake time due to poor quality of sleeping habits, which were directly associated with the timing of their first eating event. Sinha et al. [18] showed that sleep onset–wakeup and breakfast times were significantly delayed during the lockdown. Owing to the existing factors, participants had different and unfixed timing for their first meal. The inconsistency of reported timing had affected not only the Cronbach α values but also the ICC values, causing poor reliability mainly on the first eating event of weekend days.

For the existing factors that had affected the reported response, the item was later checked for the extent of the agreement by using the Bland–Altman analysis. Finally, the item was plotted on a scatter graph to describe the agreement between 2 quantitative measurements. As presented in the result part, the first eating event for weekend days with a poor Cronbach α coefficient value had a *P* value more than 0.05 for the Bland–Altman analysis, indicating the null hypothesis was rejected, an agreement was presented, and no proportional bias for the item. Hence, no changes in translation or sentence structure were made to the item. Thus, this study was able to keep all items in Malay-CPQ because they were proven to be valid and reliable. Therefore, the final version of Malay-CPQ remained with 18 items.

The reported responses of 110 respondents from both test–retest Malay-CPQ were analyzed for their chrononutrition behavior. With only slight differences in the number of reported responses, both questionnaires had similar chrononutrition behavior, preferences, and order based on scoring cutoffs for the chrononutrition profile. All respondents eventually had a good eating window (≤12:00). An eating window of <12 h is considered a good chrononutrition behavior because it allows the human body to have better digestion and be able to get a good sleep time. Moreover, excessive food intake can be avoided because the time to eat is within 12 h, which helps to avoid being overweight, obese, and cardiac disorders. A study conducted by Manoogian et al. [19] found that time-restricted feeding/eating, a new meal-timing technique that combines eating and drinking all our daily calories in a regular 8–12-h window, or less, might help with metabolic and cardiovascular health. In addition, the study stated that small human studies have shown that daily eating lengths of 4–11 h/d can lower blood pressure, improve blood sugar, and aid with weight, energy levels, sleep, and appetite.

Most respondents did not have the habit of skipping breakfast. A study by Pengpid et al. [20] found that breakfast skipping was linked to a lack of fruits and vegetables, increased soft drink consumption, and a failure to avoid fat and cholesterol. Fanelli et al. [21] supported this study, where they found that adults who did not eat breakfast showed a considerably lower overall diet quality.

An ample time given to the body to digest food is a good habit to ensure quality sleep. However, almost 80% of our respondents reported <2 h gap between the last eating event and sleep onset. It was reported previously that night-time eating was associated with additional calorie intakes among college-aged students, which supported other previous studies among shift workers, and middle older-aged populations [22]. Excessive calorie intake causes unnecessary weight gain and exposed individuals to metabolic disorders. Instead, a study by Goel et al. [23] found that evening eating did contribute to weight gain and metabolic dysfunction, regardless of calorie intake. However, a study by Maw and Haga [24] found that in middle-aged and elderly Japanese persons, 2-h or shorter intervals between dinner and night did not affect HbA1C alterations. To sustain stable HbA1C patterns, the focus should be on maintaining a normal BMI value and refraining from smoking and consuming alcohol in the long run. This finding brought new insight into which younger population who are unable to change their habit of leaving 2 h before bedtime to focus on maintaining BMI in the normal range and abstaining from smoking and drinking alcohol instead.

The reported frequency of night eating had obtained results with >80% of the respondents having a good (1 d/wk or less) score. Night-time eating is a risk factor for metabolic syndrome and obesity. This was supported by a study by Yoshida et al. [25], who found that eating behavior at night was linked to dyslipidemia in both men and women. Lunchtime was the largest meal, followed by dinner/supper and breakfast. In general, studies by Ruddick-Collins et al. [26] and Garaulet et al. [27] suggested that prioritizing energy intake earlier in the day may encourage increased energy expenditure and morning-loaded energy distribution is a viable weight-control strategy for persons who are in circadian alignment.

In summary, the chrononutrition behaviors of respondents among young adults, mainly the 6 behavior patterns, showed that they had fair to good scores (eating window, breakfast skipping, evening eating, night eating, and largest meal) except for evening latency. However, evening latency should not be an issue if the aforementioned preventive measures are followed. Moreover, the findings revealed a definite pattern in chrononutrition behaviors of undergraduate students during the COVID-19 pandemic, which health care professionals mainly might apply to improve the well-being of young adults.

The results of this study may not be representative of Malaysia because all respondents were from the Malay ethnic group, limiting its generalizability. Other limitations include that the undergraduates may have the possibility to interpret the questionnaire differently or have different meal patterns to older adults.

In conclusion, the translated Malay-CPQ version has a good content validity, face validity, and reliability. Therefore, the translated Malay-CPQ version is a reliable tool for determining the chrononutrition profile among young adults in Malaysia. However, future studies should apply the Malay-CPQ in different populations and situations in Malaysia for cross-validation because this study focused on undergraduate students during pandemic COVID-19. Thus, the Malay-CPQ version can become one of the valid indicators to measure the chrononutrition profile of the whole Malaysian community, leading to a cost-saving and effective method to assess chrononutrition behaviors for the well-being improvement of Malaysians in general.

## Data Availability

The data described in the manuscript, code book, and analytic code will be made available on request pending application.

## Funding

This work was supported by Universiti Teknologi MARA, 600-RMC/YTR/5/3 (001/2022).

## Author disclosures

All authors report no conflicts of interest.
